# Characterization of the Intestinal Microbiota of Broiler Breeders With Different Egg Laying Rate

**DOI:** 10.3389/fvets.2020.599337

**Published:** 2020-11-24

**Authors:** Zengqiao Yang, Chunhua Zhang, Jianping Wang, Pietro Celi, Xuemei Ding, Shiping Bai, Qiufeng Zeng, Xiangbing Mao, Yong Zhuo, Shengyu Xu, Hui Yan, Keying Zhang, Zhiguo Shan

**Affiliations:** ^1^Key Laboratory of Animal Disease-Resistance Nutrition, Ministry of Education, Animal Nutrition Institute, Sichuan Agricultural University, Chengdu, China; ^2^College of Agriculture and Forestry, Pu'er University, Pu'er City, China; ^3^Faculty of Veterinary and Agricultural Sciences, The University of Melbourne, Parkville, VIC, Australia

**Keywords:** broiler breeder, intestinal microbiota, ovary function, reproduction performance, laying rate

## Abstract

The gastrointestinal microbiota plays a pivotal role in maintaining animal health, immunity and reproductive performances. However, literature about the relationship between microbiota and reproductive performance is limited. The aim of the present study was to determine differences in the intestinal microbiota of broiler breeders with different egg laying rate. A total of 200 AA+ parent broiler breeders (41-week-old) were separated into two groups according to their different egg laying rate [average egg laying rate group (AR: 78.57 ± 0.20%) and high egg laying rate group (HR: 90.79 ± 0.43%). Feed conversion ratio (FCR), ovary cell apoptosis rate (ApoCR) and relative abdominal fat weight were lower (*p* = 0.01), while the hatchability rate of qualified egg was higher (*p* = 0.04) in HR group than that in AR group. *Phascolarctobacterium* abundance were lower (*p* = 0.012) in ileum of HR birds. *Romboutsia* (genus) in ileum was negatively related to the feed efficiency (*r* = −0.58, *p* < 0.05), *Firmicutes* (phylum) and *Lactobacillus* (genus) abundances in cecum were positively related to the egg laying rate (ELR) (*r* = 0.35 and 0.48, *p* < 0.05), feed efficiency (*r* = 0.42 and 0.43, *p* < 0.05), while *Spirochaetes* (phylum) and *Sphaerochaeta* (genus) abundances in cecum were negatively related to the ELR (*r* = −0.43 and −0.70, *p* < 0.05), feed efficiency (*r* = 0.54 and 0.48, *p* < 0.05), and positively related to ApoCR (*r* = 0.46 and 0.47, *p* < 0.05). Our results suggested that microbiota, such as *Firmicutes* (phylum) and *Lactobacillus* (genus) have positive relationship, while *Spirochaetes* (phylum) *and Romboutsia* (genus) abundances exert negative relationship with broiler breeders' reproductive performances.

## Introduction

Poultry meat is one of the most important sources of protein (meat and eggs) for human nutrition (1). Over the past years, a significant improvement in poultry production has resulted in extremely high level of efficiency in both broiler chickens and laying hens, with birds reaching 3 kg in body weight at 40 days of age and hens laying 500 eggs in 100 weeks (2). The microbiota is defined as the microbial community, including commensal, symbiotic and pathogenic microorganisms (3) which could interact with the host, resulting in the influence on metabolism, immunity and even behavior (4, 5). A normal and stable microbiota play a pivotal role in maintaining optimal gastrointestinal functionality, animal health, welfare and production performances (6). Accumulating evidences suggest that the intestinal microbiota could affect the poultry production (7, 8). The gastrointestinal (GIT) microbiota contributes to the regulation of fat deposition which in poultry seems to be independent of host genetics (9). These observations are not surprising considering that microbial metabolites such as short-chain fatty acids have been implicated in the modulation of energy metabolism (10). Furthermore, the microbiota can improve feed conversion efficiency as a consequence of their ability to synthesize beneficial nutrients such as vitamins (11, 12) and by improving the energy harvest from the diet, resulting in improved growth performances. Moreover, in addition to the well-known links between the GIT microbiota and the neuroendocrine function of the gut (13, 14), it has been shown that dysbiosis of the GIT microbiota can result in the activation of the immune system which in turn raise serum insulin levels and androgen production disrupting ovarian function (15). Indeed, in humans, polycystic ovary syndrome (PCOS) is associated with altered insulin sensitivity and hormonal imbalance (16). However, whether the GIT microbiota is involved in the regulation of ovarian activity in poultry is still not known.

In this study, we intended to explore the possible relationships between the GIT microbiota and ovarian function in broiler breeders with different egg laying rate.

## Materials and Methods

### Birds, Experimental Design and Management

After the Pre-Test (Supplementary Materials and Method), a total of 41-week-old 200 AA+ parent breeders (live weight: AR: 4.08 ± 0.12 vs. HR:4.15 ± 0.07 kg) from the same flock and the same house were divided into two separate groups, according to their egg laying rate (ELR; AR: 78.57 ± 0.20% vs. HR: 90.79 ± 0.43%) (Supplementary Materials and Method). For each of the two groups 10 replicates of 10 birds each were enrolled in a 42-days trial. Breeders were subjected to artificial insemination every 4 days. All birds were fed restrictedly with the same diet (Supplementary Table 1) for about 162 gram per bird per day in order to avoiding the difference brought by feed consumption. Environmental temperature was maintained at 22 ± 1°C; the daily lighting schedule was 16 h light and 8 h dark. Birds had *ad libitum* access to water. This study was approved by the guidelines (SYXK2014-187) of the Animal Care and Use Committee of Sichuan Agricultural University and meets the guidelines set by the Regulations for the Administration of Affairs Concerning Experimental Animals of the State Council of the People's Republic of China.

### Sample Collection and Measurements

Egg production, broiler breeder mortality, qualified egg (Except for: egg weight < 50 g or >75 g, misshaped egg, dirty egg, and sand-shelled egg) and feed consumption were recorded daily. The feed conversion ratio (FCR) was calculated accordingly. Hatchability was recorded at day 42. At end of the study, birds (20 birds/treatment) were sacrificed by CO_2_ suffocation and the ovary and intestines were removed for the measurement of relative weight and length. The content of the duodenum, jejunum, ileum and cecum was immediately transferred into 1.5 ml sterile centrifuge tubes for microbiota analysis. The ovaries were collected and placed into 4% paraformaldehyde (pH = 7.2) for cell apoptosis analysis.

### Ovary Apoptosis by TUNEL Method

The ovary was quickly removed and placed into immediately into methyl aldehyde, then were histochemical stained using TUNEL technique by an *in situ* apoptosis detection kit (*in situ* cell death detection kit-POD, Roche Group, Switzerland). Using BA200Digital (Mike Audi Industrial Group Co., Ltd.) to image acquisition. Apoptotic color is light yellow or brown yellow, and negative expression is blue with white background. Totally, 100 images have been taken to measure the cell apoptosis, and apoptosis rate is defined as the percentage of apoptotic cells in 100 cells counted.

### DNA Extraction and Microbiota Analysis

Total DNA was extracted from the chyme of duodenum, jejunum, ileum and cecum, using the TIANamp Bacteria DNA isolation kit (DP302-02, Tiangen, Beijing, China) according to the manufacturer's instructions. 16S rRNA genes of distinct regions (16S V4) were amplified. All PCR reactions were carried out with Phusion® High-Fidelity PCR Master Mix (New England Biolabs). Operate electrophoresis on 2% agarose gel to detect, PCR products, a bright main strip between 300–400 bp, were chosen. Then PCR products were mixed in equal density ratios. Then mixture PCR products was purified with Qiagen Gel Extraction Kit (Qiagen, Germany). Sequencing libraries were generated via the TruSeq®DNA PCR-Free Sample Preparation Kit (Illumina, USA) following manufacturer's recommendations, index codes were added. Finally, the library was sequenced on an IlluminaHiSeq2500 platform and 250 bp paired-end reads were generated. Paired-end reads were assigned to samples based on their unique barcode and truncated by cutting off the barcode and primer sequence. Paired-end reads were merged by using FLASH (Version 1.2.7), and then quality filtration was using QIIME (Version 1.7.0) to finish. Chimera sequences were removed using the UCHIME algorithm (17). Sequence clustering to generate operational taxonomic units (OTUs) with ≥ 97% similarity was performed via UPARSE.

The representative sequences of each OTU were aligned to the Silva Database(Version 128) based on the RDP classifier (Version 2.2) algorithm to annotate taxonomic information (18). Alpha diversity metrics (Observed-species, Chao1, Shannon, Simpson, ACE, and Good-coverage) were calculated with QIIME (Version 1.7.0) and displayed with R software (Version 2.15.3). Beta diversity metrics (weighted UniFrac and unweighted UniFrac) were calculated with QIIME software (Version 1.7.0). PCoA analysis based on weight-unifrac was conducted with the WGCNA package, stat packages, and the ggplot2 package in R software (Version 2.15.3). Spearman analysis was conducted with the diversity/alpha diversity index package and the environmental factors package in R software. The sequence data reported in this study have been deposited in the NCBI database (http://www.ncbi.nlm.nih.gov/bioproject/663043, accession No. is PRJNA663043).

### Statistical Analysis

Data of performance parameters was analyzed by independent-sample *T*-test in the SPSS version 25.0 statistical software package (IBM® SPSS® Statistics, New York, USA), differences among treatments were considered significant at *p* < 0.05. Each pen served as the experimental unit. For the microbiota data, alpha indexes were analyzed by Wilcox rank sum test, differences among treatments were considered significant at *p* < 0.05. Differences of relative abundance levels of the bacteria was analyzed by *T*-test, differences among treatments were considered significant at *p* < 0.05. AMOVA test was based on unweighted UniFrac distance, differences among treatments were considered significant at *p* < 0.05.

## Results

### Reproductive Performance in Different Egg Laying Rate Breeders

There were no differences in average egg weight and qualified egg rates were observed between the two groups (Supplementary Table 2). Egg laying rate (*p* < 0.01) and hatchability of qualified egg rate (HQR; *p* = 0.04) were higher in HR group, as expected, while FCR was lower (*p* < 0.01) in the HR birds.

### Gastrointestinal Organs Weight in Different Egg Laying Rate Breeders

Compared to the AR groups, birds in the HR group presented a lower abdominal fat content (*p* = 0.01), however no differences were observed for chicken weight, relative weight of crop, proventriculus, gizzard, intestines (duodenum, jejunum, ileum, cecum) (Supplementary Table 3) and length of intestines (Supplementary Table 4).

### Ovary Cell Apoptosis in Different Egg Laying Rate Breeders

**Figure 1:**
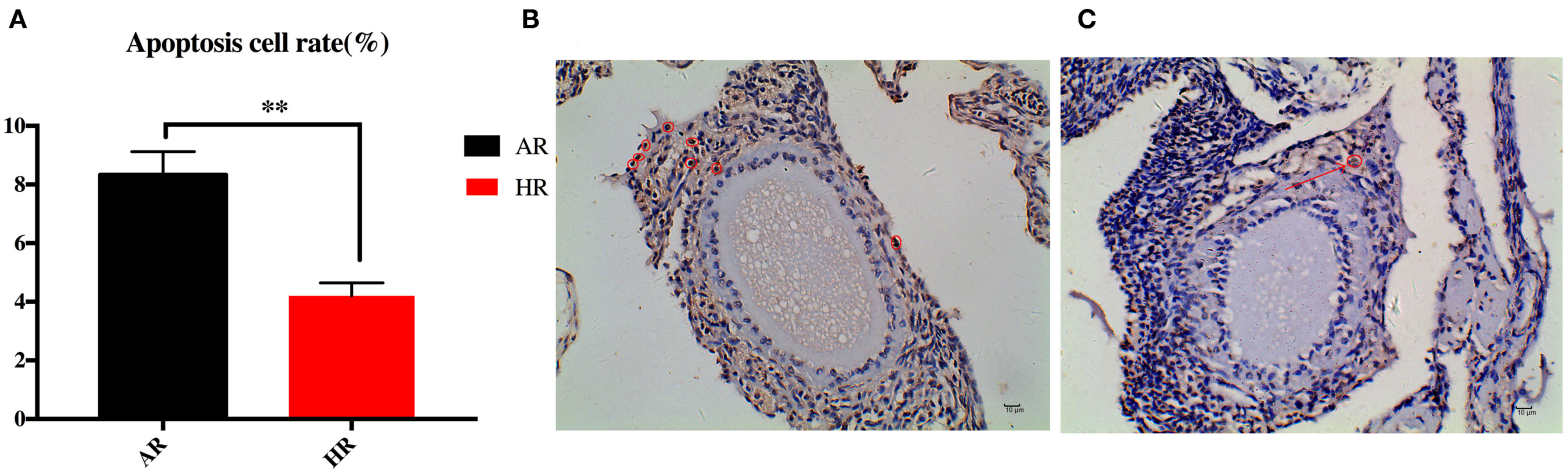
Differences in ovary apoptosis rate **(A)** between AR and HR broiler breeders. Ovary of AR **(B)** and HR **(C)** birds; apoptotic cells are marked with red circle (*n* = 10); ** indicted significant difference between two groups (*P* < 0.01).

As shown in Figure 1, cell apoptosis rate (ApoCR) in the ovaries of the HR birds was lower than that observed in the AR group (*p* < 0.01).

### Microbiota Composition in Broiler Breeder With Different Egg Laying Rate

**Figure 2:**
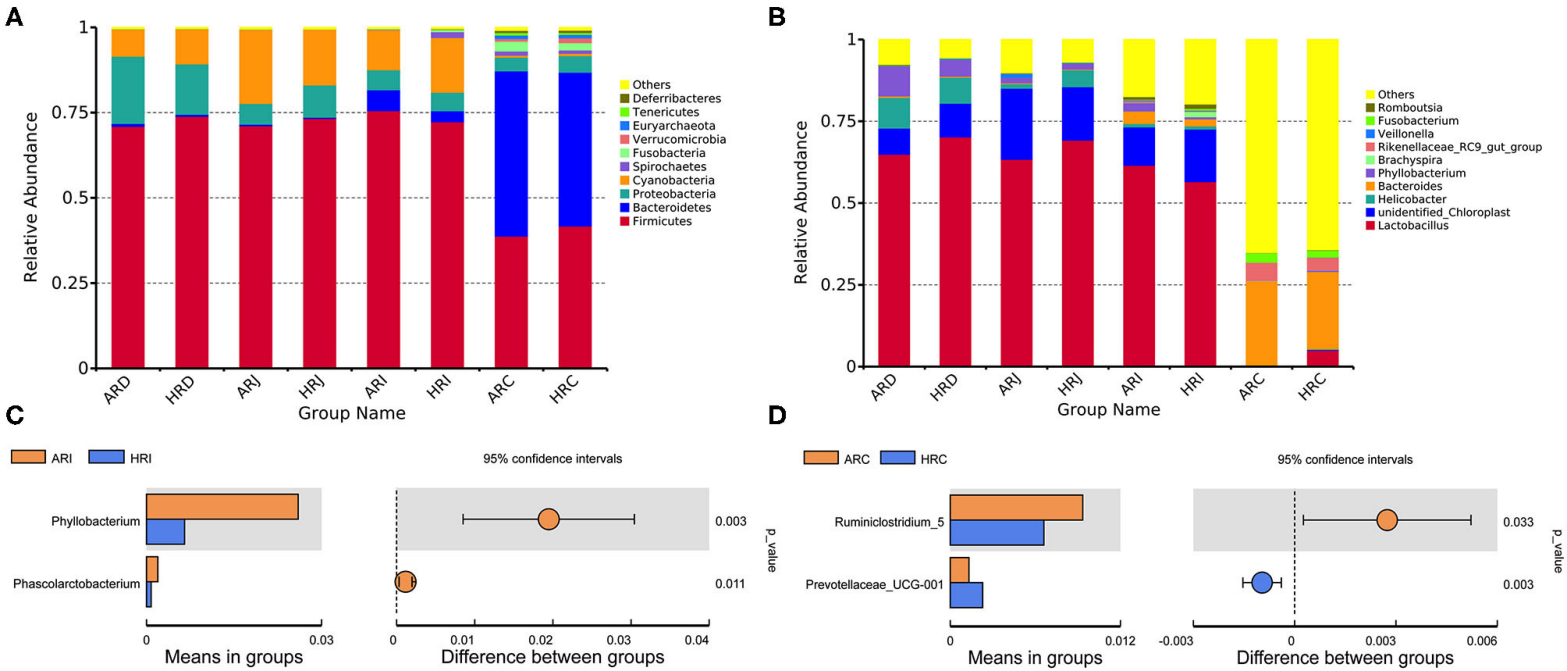
Differences in richness **(A,B)**, diversity **(C,D)** index in the duodenum (AR.D), jejunum (AR.J), ileum (AR.I), cecum (AR.C) of AR and the duodenum (HR.D), jejunum (HR.J), ileum (HR.I), and cecum (HR.C) of HR (*n* = 8).

The present study obtained a total of 5,328,583 high quality 16S rRNA gene sequences from duodenum (1,253,578), jejunum (1,320,806), ileum (1,347,555), cecum (1,406,644), with an average of 83,259 sequences per sample and a range of 67,290–95,053. A total of 1,472 OTUs were identified at 97% sequence identity. A total top 10 phyla and top 10 genera were based on the identified OTUs. At phylum level, *Firmicutes, Proteobacteria* in duodenum, *Firmicutes* in jejunum and ileum, *Firmicutes, Bacteroidetes* in cecum were the predominant phyla (Figure 2A) (Table 1). At genus level, *Lactobacillus, Unidentified_Chloroplast, Helicobacter* and *Bacteroides* in duodenum, jejumum and ileum, *Lactobacillus, Fusobacterium, Bacteroides*, and *Rikenellaceae* in cecum were the predominant genera (Figure 2B) (Table 2). With the *T*-test, in ileum *Pyhllobacterium* (*p* < 0.01) and *Phascolarctobacterium* abundances were lower (*p* = 0.012) (Figure 2C) in HR, in cecum *Ruminiclostridium_5* abundance was lower (*p* = 0.032) and *Precotellaceae_UCG-001* abundance was higher (*p* < 0.01) (Figure 2D) in HR.

**Table 1:** Differences in the relative abundance of dominant microbiota (phylum) ratio in different intestinal segments between different egg laying rate broiler breeders (%) (*n* = 8)^a^.

**Phylum**	**Group**	**Firmicutes**	**Bacteroidetes**	**Fusobacteria**	**Proteobacteria**	**Cyanobacteria**	**Spirochaetes**	**Verrucomicrobia**	**Others**
Duodenum	AR	70.97	0.85	0.09	19.73	7.96	0.02	0.01	0.38
	HR	73.92	0.57	0.08	14.78	10.28	0.01	0.01	0.35
*P*-value		0.71	0.50	0.44	0.55	0.65	0.86	0.88	0.54
Jejunum	AR	71.12	0.48	0.05	6.08	21.73	0.01	0.01	0.51
	HR	73.30	0.38	0.04å	9.42	16.34	0.01	0.01	0.50
*P*-value		0.79	0.76	0.25	0.49	0.63	0.93	0.20	0.90
Ileum	AR	75.58	6.12	0.15	5.88	11.73	0.14	0.01	0.36
	HR	72.28	3.24	0.57	5.43	16.07	1.72	0.06	0.52
*P*-value		0.68	0.58	0.69	0.46	0.38	0.29	0.27	0.27
Cecum	AR	38.79	48.44	2.90	4.02	0.61	1.21	0.74	3.23
	HR	41.76	45.09	2.15	4.85	0.68	0.99	1.42	1.35
*P*-value		0.21	0.20	0.12	0.82	0.48	0.54	0.37	0.62

a *Each mean represents 10 replicates, with one layer/replicate. Abbreviation represents: AR, average egg laying rate; HR, high egg laying rate*.

**Table 2:** Differences in the relative abundance of dominant microbiota (genus) ratio in different intestinal segments between different egg laying rate broiler breeders (%) (*n* = 8)^a^.

**Genus**	**Group**	**Lactobacillus**	**Fusobacterium**	***Unidentified_chloroplast***	**Helicobacter**	**Bacteroides**	**Phyllobacterium**	**Romboutsia**	**Others**
Duodenum	AR	64.93	0.08	7.95	9.38	0.48	9.15	0.23	7.80
	HR	70.22	0.08	10.28	7.95	0.33	5.08	0.09	5.98
*P*-value		0.50	0.55	0.81	0.53	0.16	0.86	0.12	0.40
Jejunum	AR	63.37	0.05	21.72	1.37	0.27	1.62	0.17	11.44
	HR	69.22	0.05	16.34	5.17	0.23	1.70	0.09	7.21
*P*-value		0.49	0.49	0.22	0.82	0.88	0.92	0.21	0.07
Ileum	AR	61.54	0.15	11.72	1.05	3.82	2.60	0.82	18.30
	HR	56.54	0.57	16.05	0.97	2.19	0.65	1.31	21.73
*P*-value		0.67	0.46	0.84	0.63	0.00	0.29	0.65	0.67
Cecum	AR	0.41	2.90	0.03	0.05	25.65	0.03	0.07	70.86
	HR	5.00	2.15	0.20	0.25	23.64	0.43	0.13	68.20
*P*-value		0.17	0.22	0.15	0.45	0.18	0.54	0.57	0.57

a *Each mean represents 10 replicates, with one layer/replicate. Abbreviation represents: AR, average egg laying rate; HR, high egg laying rate*.

### Alpha Diversity of Microbiota in Different Egg Laying Rate Breeders

**Figure 3:**
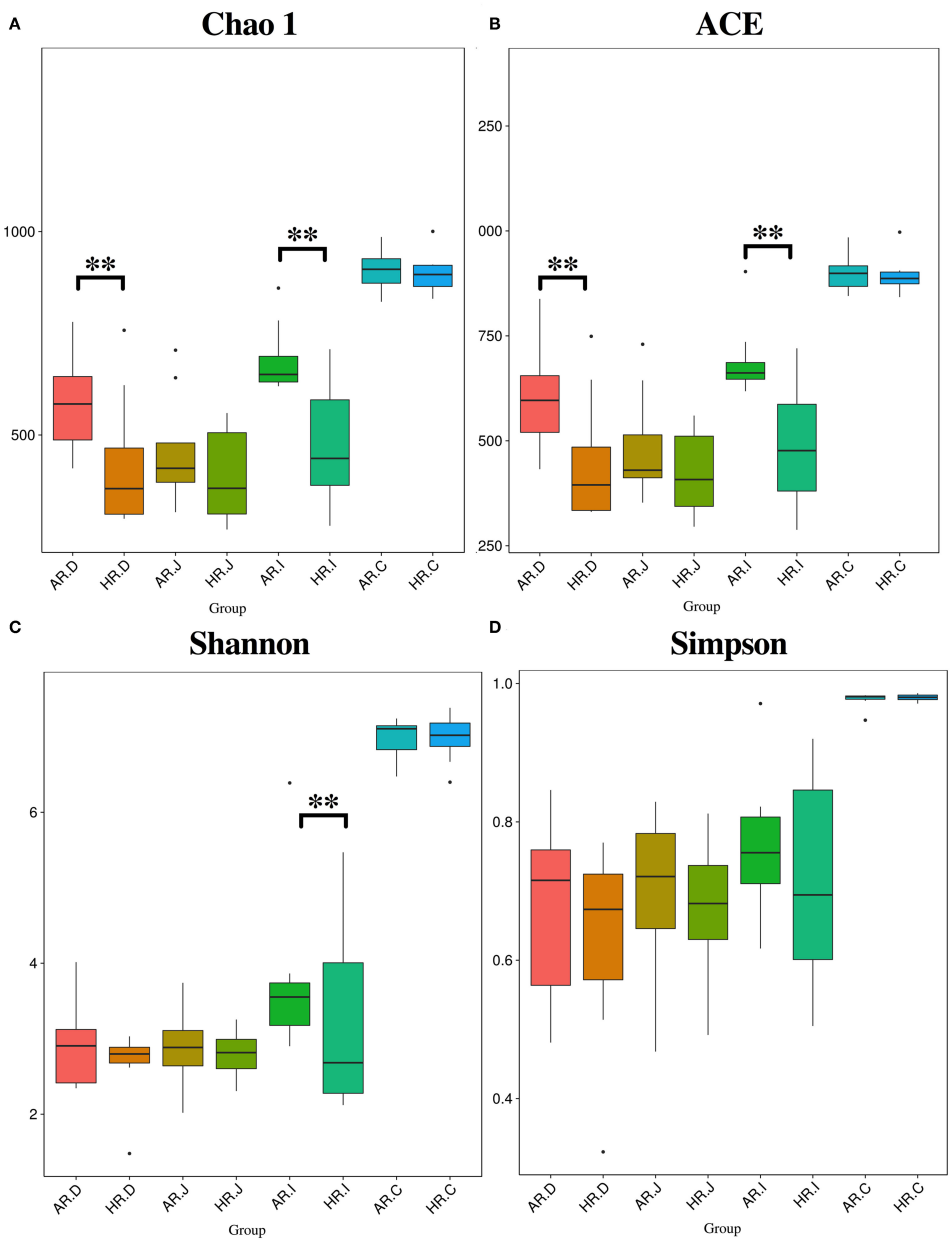
Differnces in relative abundance levels of the bacterial phyla **(A)** and genera **(B)** in the duodenum (AR.D), jejunum (AR.J), ileum (AR.I), cecum (AR.C) of AR and the duodenum (HR.D), jejunum (HR.J), ileum (HR.I), cecum (HR.C) of HR. *T*-test based on the relative abundance levels of the bacterial. *T*-test on ileal bacteria **(C)**, caecal bacteria **(D)** at genus level (*n* = 8); ** indicted significant difference between two groups (*P* < 0.01).

The Chao1 and ACE indexes were lower in duodenum and ileum of HR (*p* < 0.01) (Figures 3A,B) and Shannon index was lower in ileum of HR (*p* < 0.01) (Figure 3C). The Venn and Flower diagrams were used to describe common and unique OTUs between goups. In the duodenum, jejunum, ileum and cecum of the AR and HR birds, we observed, the common OTUs is 713, 595, 756, and 1,046, respectively (Supplementary Figure 1).

### Beta Diversity of Microbiota in Different Egg Laying Rate Breeders

**Figure 4:**
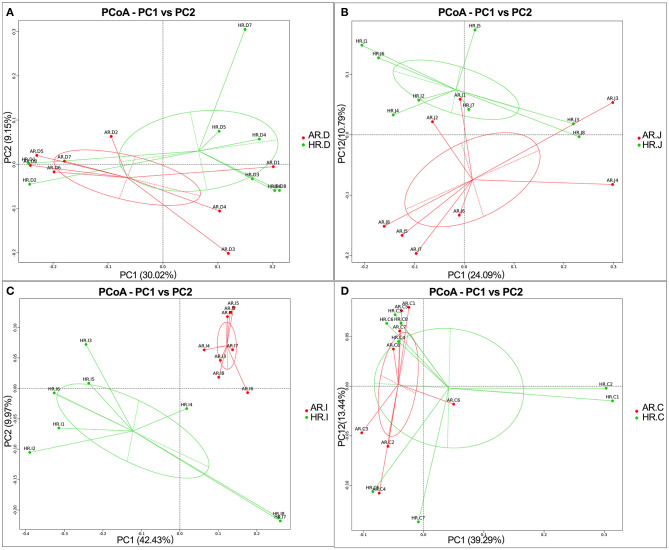
Principal coordinate analysis (PCoA) of microbial comunities in the duodenum **(A)**, jejunum **(B)**, ileum **(C)**, cecum **(D)**, of AR and HR broiler breeders (*n* = 8).

As is shown in Figure 4, microbiota communities of duodenum, jejunum, ileum and cecum based on unweighted UniFrac distances with OTUs indicated that microbiota of AR and HR each formed a distinct cluster among all samples, and these clusters were separated from each other. Moreover, AMOVA results indicated that there was significant difference in bacterial communities in ileum of AR and HR (*p* = 0.01) (Supplementary Table 5).

### Correlation Between Performance and Microbiota

As is shown in Table 3, 4, spearman correlation analysis was employed to explore correlations between microbiota and performance parameters (ELR, FCR, HQR) and ovary function (ApoCR) measured in this study. It has shown that *Romboutsia* (genus) abundance in ileum was negatively related to the feed efficiency (*r* = −0.58, *p* < 0.05), *Firmicutes* (phylum) and *Lactobacillus* (genus) abundances in cecum were positively related to the ELR (*r* = 0.35 and 0.48, *p* < 0.05), feed efficiency (*r* = 0.42 and 0.43, *p* < 0.05), while *Spirochaetes* (phylum) and *Sphaerochaeta* (genus) abundances in cecum were negatively related to the ELR (*r* = −0.43 and −0.70, *p* < 0.05), feed efficiency (*r* = 0.54 and 0.48, *p* < 0.05), and positively related to ApoCR (*r* = 0.46 and 0.47, *p* < 0.05).

**Table 3:** The correlation between dominant bacteria in the ileum with broiler breeders' reproductive performance (r).

**Items**	**Microbiota**	**Reproductive performance**			**Ovary apoptosis**
		**ELR**	**FCR**	**HQR**	**ApoCR**
Phylum	Firmicutes	0.12	−0.29	0.10	−0.23
	Cyanobacteria	0.00	0.02	0.22	−0.10
	Bacteroidetes	−0.28	0.33	−0.26	0.41
	Spirochaetes	−0.33	0.33	−0.35	0.27
	Proteobacteria	−0.26	0.28	0.01	0.25
	Fusobacteria	−0.35	0.26	−0.28	0.48
	Deferribacteres	−0.27	0.35	−0.22	0.30
	Tenericutes	−0.20	0.37	−0.39	0.13
	Verrucomicrobia	0.04	−0.03	0.17	0.02
	Euryarchaeota	−0.61*	0.58*	−0.37	0.73*
Genus	*Lactobacillus*	−0.02	−0.11	0.23	−0.19
	*Unidentified_Chloroplast*	0.00	0.02	0.22	−0.10
	*Bacteroides*	−0.25	0.28	−0.26	0.39
	*Brachyspira*	−0.08	0.18	−0.30	0.08
	*Romboutsia*	−0.46	0.58*	−0.25	0.41
	*Phyllobacterium*	−0.77	0.62	−0.38	0.33
	Rikenellaceae_RC9_gut_group	−0.32	0.43	−0.21	0.45
	*Helicobacter*	0.36	−0.13	0.15	−0.31
	*Fusobacterium*	−0.35	0.26	−0.28	0.48
	*Veillonella*	−0.50	0.28	−0.09	0.24

**Table 4:** The correlation between dominant bacteria in the cecum with broiler breeders' reproductive performance (r).

**Items**	**Microbiota**	**Reproductive performance**			**Ovary apoptosis**
		**ELR**	**FCR**	**HQR**	**ApoCR**
Phylum	Bacteroidetes	−0.34	0.29	−0.15	0.23
	Firmicutes	0.35*	−0.42*	0.05	−0.24
	Fusobacteria	−0.15	−0.05	0.14	0.16
	Proteobacteria	0.06	−0.01	0.29	−0.02
	Verrucomicrobia	−0.09	0.18	−0.07	0.09
	Spirochaetes	−0.43*	0.54*	−0.08	0.46*
	Euryarchaeota	0.03	−0.05	−0.02	0.03
	Tenericutes	−0.01	−0.09	−0.18	−0.22
	Cyanobacteria	0.13	−0.04	−0.10	−0.01
	Planctomycetes	−0.25	0.31	−0.62	0.42
Genus	*Bacteroides*	−0.32	0.00	−0.34	0.20
	*Lactobacillus*	0.48*	−0.43*	0.04	−0.33*
	*Fusobacterium*	−0.15	−0.05	0.14	0.16
	*Methanobrevibacter*	0.16	−0.21	0.01	0.02
	*Alloprevotella*	−0.08	< 0.01	−0.48	0.01
	*Tyzzerella*	0.07	−0.02	0.04	−0.08
	Ruminococcaceae_NK4A214_group	−0.44	0.38	−0.17	0.07
	*Helicobacter*	0.32	−0.14	0.02	−0.18
	*Sphaerochaeta*	−0.70*	0.48*	−0.35*	0.47*
	*Parabacteroides*	−0.45	0.30	−0.21	0.32

*Abbreviation represents: ELR, egg laying rate; FCR, feed conversion ratio; ApoCR, ovary apoptosis cell rate*.

## Discussion

The main function of the GIT is to digest feed and absorb nutrients. In the small intestine (duodenum, jejunum and ileum), the host absorbs mostly the nutrients of dietary origin (19), while in the cecum, the host absorbs nutrients produced by microbial fermentation (20, 21). It is well-accepted that productive and reproductive performances are influenced by absorption and utilization of nutrients in poultry. In the present study, even though both AR and HR birds were fed the same diet, we observed that abdominal fat was lower in the HR birds (the abdominal fat rate of two experimental groups is different at the beginning of the experiment), but both body and organ weight was not different from that of AR birds and FCR was lower in HR birds. That indicated nutrients requirement of two experimental groups were different, so HR group needed more energy because they produce more eggs and had better energy utilization. What's more, the abundance of *Romboutsia* (genus), a relatively higher abundance of bacteria in the ileum of HR birds, was positively correlated with FCR. Analysis to *Romboutsia ilealis* of the genome in the small intestinal tract revealed it had the ability to use carbohydrates. And multiple and partially redundant pathways for utilization of a wide array of relatively simple carbohydrates be identified, also, a whole-genome transcriptome analysis pinpointed components of the key pathways involved in the degradation of glucose, L-fucose and fructo-oligosaccharide in mice (22). What's more, *Romboutsia sedimentorum* abundances after utilizing glucose generated end products like acetic and iso-butanoic acids which were beneficial to reducing obesity (23) Therefore, in present study, the higher abundance of *Romboutsia* observed in the HR birds might be linked to the lower abdominal fat in HR birds, which suggests that energy and nutrients were not being diverted to abdominal fat deposit as observed in the HR birds. The fatty acid-induced cytotoxicity, namely lipotoxicity, of pancreatic β cells is associated with increased cellular palmitoyl-CoA that promotes ceramide accumulation and cell apoptosis (24). It has been reported that changes in ovarian function are accompanied by increased abdominal adiposity (25, 26) and that body fat is negatively correlated with fertility (27). These observations support our findings that ApoCR was higher in the AR birds.

The nutritional requirements of the gastrointestinal microbiota are species-dependent as bacteria need different dietary substrates for their growth and metabolic activity (21). In the present study, we observed that the duodenum and jejunum microbiota of AR birds have more richness than that of the HR ones, and the ileum microbiota was more diverse in the AR birds than in the HR ones. It is generally accepted that higher diversity and richness of gut bacteria is beneficial for host gut health, but higher microbe abundance could have resulted in a nutrients competition between the host and the microbes in small intestine because they use the same nutrient (carbohydrates, protein, etc.) as the substrate for proliferation (8, 21, 28). The lower feed efficiency (higher FCR) observed in the AR birds could support this hypothesis. In the present study, *Firmicutes* and *Proteobacteria* were the dominant phyla in the small intestines while *Bacteroidetes* was the dominant bacteria in the cecum. *Lactobacillus* was the dominant genus in the small intestines while *Bacteroides* was the dominant genus in the cecum. Similar observations have been made in broiler chickens and laying hens (29, 30). Diet is a well-known factor that can influence the microbiota composition and metabolic activity (31, 32). For example, an altered abundance of *Bacteriodetes* and *Firmicutes* abundances has been observed in animals fed a high fat diet (33, 34). Indeed, in present study, there was significant differences of bacterial communities in ileac tract of AR and HR with AMOVA test, while those in rest of tract of AR and HR were no significant.

It is worth noting that we observed a higher relative abundance of *Phascolarctobacterium* in the ileum of AR birds. A higher abundance of *Phascolarctobacterium* has been correlated with elevated oxidative stress in sows during late gestation and at parturition (35) and intestinal inflammation and pathology in humans (36). Finally, *Phascolarctobacterium* abundance seems to be correlated with increased body weight and fat mass, and altered metabolic profile (37). However, the abundance of *Phascolarctobacterium* in AR birds was not prevalence (comparative low, about 0.3%), further metabolite pathways analysis was needed to determine the efficiency of the *Phascolarctobacterium* in current study.

In our study we observed that *Firmicutes* (phylum) and *Lactobacillus* (genus) abundances in cecum were positively related to the reproductive performance (higher ELR, feed efficnecy, lower ApoCR). The *Firmicutes/Bacteroidetes* ratio is considered a biomarker of gastrointestinal functionality and can be indicative of eubiosis conditions in the gastrointestinal tract (38). It has been indicated that *Firmicutes* such as *Lactobacillus* and *Lactococcus spp*. have biotechnological value in fermentation and bacteriocin production, which may benefit the host and increase the reproductive performance. *Spirochaetes* (phylum) abundance in the cecum was positively correlated with FCR. *Spirochaetes* are responsible for infectious typhlitis in hens which is characterized by decreased egg production and increased the feed consumption (39). Broiler breeders infected by *Spirochaetes* had poorer feed conversion and the impact of the disease was also extended to their offspring, with chicks being weak, slow growing and with impaired gastrointestinal functionality (40). In this study, the lower productive and reproductive performances observed in the AR birds could be partially ascribed to the higher relative abundance of *Spirochaetes* in the cecum of the AR birds.

## Conclusions

Overall, our findings suggest that higher diversity and richness of microbial communities in the small intestines negatively impact with reproduction. What's more, the higher relative abundance of *Romboutsia* (genus) and *Spirochaetes* (phylum) in the AR birds seems to be correlated to their lower reproduction performances, while the higher richness of *Firmicutes* (phylum) and *Lactobacillus* (genus) in cecum may benefit the reproductive performance of breeders.

## Implication

A normal and stable microbiota play a pivotal role in maintaining optimal gastrointestinal functionality, animal health, welfare and production performances. In this study, we intended to explore the possible relationships between the GIT microbiota and ovarian function in broiler breeders with different egg laying rate. Our results suggest that microbiota, such as *Firmicutes* (phylum) and *Lactobacillus* (genus) have positive relationship, while *Spirochaetes* (phylum) *and Romboutsia* (genus) exert negative relationship with broiler breeders' reproductive performances. This study reveals one potential relationship between production performance and microbiota in poultry.

## Data Availability Statement

The datasets generated for this study can be found in NCBI BioProject, NCBI Accession No. PRJNA663043.

## Ethics Statement

The animal study was reviewed and approved by the guidelines (SYXK2014-187) of the Animal Care and Use Committee of Sichuan Agricultural University and meets the guidelines set by the Regulations for the Administration of Affairs Concerning Experimental Animals of the State Council of the People's Republic of China.

## Author Contributions

JW, ZS, and KZ conceived and designed the experiments. JW, ZY, XD, and SB performed the experiments. JW, ZY, and QZ analyzed the data. ZY and JW wrote the paper. PC, ZY, XM, HY, CZ, and XD helped revise this manuscript. All authors read and approved the final manuscript.

## Conflict of Interest

The authors declare that the research was conducted in the absence of any commercial or financial relationships that could be construed as a potential conflict of interest. The handling Editor declared a past collaboration with the authors JW and XM.
