# Supplementary material for: Characterization of the Intestinal Microbiota of Broiler Breeders With Different Egg Laying Rate
Source: Front Vet Sci. 2020 Nov 24;7:599337. doi: 10.3389/fvets.2020.599337 (PMC7732610; doi:10.3389/fvets.2020.599337)
Supplement: Supplementary file 1 [file Data_Sheet_1.docx]

Supplementary Material

# Supplementary Material and Methods

*Birds, experimental design and Management*

Pre-Test: In the pre-test, we recorded the egg laying rate of 5000 AA+ parent broiler breeders (36-week-old) that were fed restrictedly with the same diet (Supplementary Table S1) for about 162 gram per bird per day and had *ad libitum* access to water, in the same house at the temperature of 20℃±1℃; and the 16 hours light and 8 hours dark of daily lighting schedule for 2 weeks. What's more, every bird was numbered, so we could track everyone's production. According to their laying rate, we chose 200 birds (100 birds' laying rate is 78.57%±0.20% named "AR", the left 100 birds' laying rate is 90.79%±0.43% named "HR"), and put AR birds into new cages with 2 birds per cage, and HR birds were to do so. The AR cage is set aside by the HR cage one by one. Additionally, birds were subjected to artificial insemination every 4 days just like the manipulation schedule before. And in order to make sure them could suit for new cages to avoid stress, we fed them for another 3 weeks. To sum up, the only changed set is the cage in Pre-Test. Also, we checked the health and comfort of all birds everyday for the formal trial.

Formal trial: As described in "Manuscript", and the fed diet of 162 gram per bird per day was eaten by a bird with no left.

# Supplementary Figures and Tables

## Supplementary Tables

| **Supplementary Table S1**. Composition and nutrient levels of basal diet | | | |
| --- | --- | --- | --- |
| Item | Content (%) | Calculated nutrient content (%) | |
| Corn | 69.50 | ME（MJ/kg) | 11.61 |
| Soybean meal (43% CP) | 19.00 | CP | 13.8 |
| Soybean oil | 1.00 | Ca | 3.4 |
| Calcium Carbonate | 8.25 | AP | 0.3 |
| Calcium Hydrophophate | 1.14 | DLys | 0.74 |
| L-Lysine HCl | 0.08 | DMet | 0.34 |
| DL-Methionine | 0.11 | Met-Cys | 0.59 |
| Threonine | 0.02 | DThr | 0.54 |
| Sodium chloride | 0.30 |  |  |
| Choline chloride,50% | 0.10 |  |  |
| Vitamin and mineral premix^1^ | 0.50 |  |  |
| Total | 100 |  |  |
| ^1^Provide per kilogram of diet: VA 12000 IU; VD_3_ 4000 IU; VK_3_ 4.0 mg; VB_1_ 3.0 mg; VB_2_ 11.5 mg; VB_6_ 7.0 mg; VB_12_ 0.02 mg; folic acid 8.6 mg; nicotinic acid 47.2 mg; pantothenic acid 21.7 mg; biotin 0.6 mg; Fe 95 mg; Cu 25 mg; Mn 100 mg; Zn 100 mg; Se 0.45 mg; I 1.4 mg. | | | |

| **Supplementary Table S2**. Difference in productive performance between different egg laying rate broiler breeders (n=10)^1^ | | | | | |
| --- | --- | --- | --- | --- | --- |
| Item | Egg laying rate, % | Average egg weight, g | FCR | Qualified egg rate, % | Hatchability rate, % |
| AR | 77.26 | 66.39 | 3.16 | 90.86 | 84.17 |
| HR | 86.67 | 64.15 | 2.92 | 93.80 | 98.33 |
| SEM | 0.008 | 0.805 | 0.042 | 0.029 | 0.411 |
| P-Value | <0.01 | 0.07 | <0.01 | 0.20 | 0.04 |
| ^1^Each mean represents 10 replicates, with one layer/replicate. | | | | | |

| **Supplementary Table S3**. Difference in relative gastrointestinal organ weight between different egg laying rate broiler breeders (n=10) ^1^ | | | | | | | | | |
| --- | --- | --- | --- | --- | --- | --- | --- | --- | --- |
| Item | Live weight, kg | Crop (%) | Proventriculus (%) | Abdominal fat (%) | Gizzard (%) | Duodenum (%) | Jejunum (%) | Ileum (%) | Cecum (%) |
| AR | 4.08 | 0.35 | 0.21 | 2.99 | 0.84 | 0.29 | 0.45 | 0.39 | 0.23 |
| HR | 4.15 | 0.40 | 0.20 | 2.36 | 0.88 | 0.27 | 0.46 | 0.36 | 0.22 |
| SEM | 0.089 | <0.01 | <0.01 | 0.21 | <0.01 | <0.01 | <0.01 | <0.01 | <0.01 |
| P-value | 0.59 | 0.11 | 0.8 | 0.01 | 0.26 | 0.57 | 0.82 | 0.45 | 0.88 |
| ^1^Each mean represents 10 replicates, with one layer/replicate. Abbreviation represents: AR = average egg laying rate, HR = high egg laying rate. | | | | | | | | | |

| **Supplementary Table S4**. Differences in gastrointestinal organ length between different egg laying rate broiler breeders (n=10) | | | | |
| --- | --- | --- | --- | --- |
| Item | Duodenum (cm) | Jejunum (cm) | Ileum (cm) | Cecum (cm) |
| AR | 34.87 | 79.66 | 80.24 | 43.96 |
| HR | 36.84 | 86.35 | 76.74 | 41.18 |
| SEM | 0.880 | 2.945 | 3.229 | 1.601 |
| P-Value | 0.14 | 0.14 | 0.49 | 0.24 |
| ^11^Each mean represents 10 replicates, with one layer/replicate. Abbreviation represents: AR = average egg laying rate, HR = high egg laying rate. | | | | |

| **Supplementary Table S5**. Differences between groups of AMOVA analysis for different egg laying rate broiler breeders (n=8) | | | | | |
| --- | --- | --- | --- | --- | --- |
| vs_group | SS | df | MS | Fs | p-value |
| ARD-HRD | 0.151499(1.73109) | 1(14) | 0.151499(0.123649) | 1.22524 | 0.179 |
| ARJ-HRJ | 0.143406(1.60301) | 1(14) | 0.143406(0.114501) | 1.25244 | 0.167 |
| ARI-HRI | 0.325502(1.40049) | 1(14) | 0.325502(0.100035) | 3.25388 | 0.011 |
| ARC-HRC | 0.0480475(0.539249) | 1(14) | 0.0480475(0.0385178) | 1.24741 | 0.311 |

## Supplementary Figures


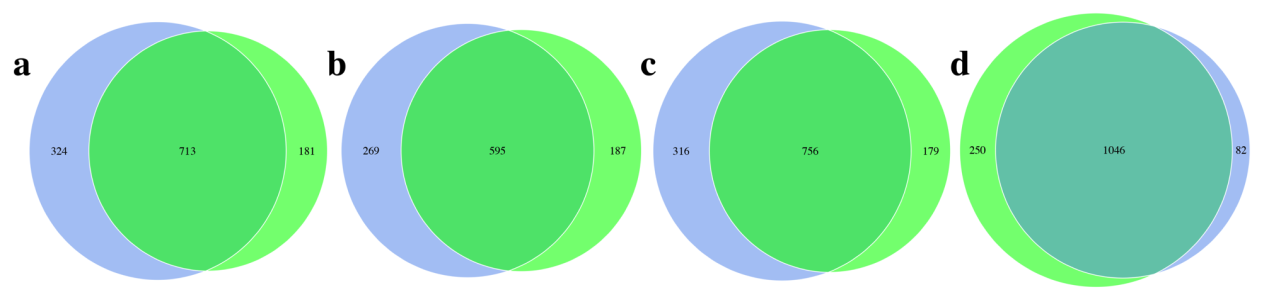


**Supplementary Figure 1.** Differences in OTUs distribution. The Venn diagrams are used to describe common and unique OTUs between goups. duodenum AR-HR (**a**), jejunum AR-HR (**b**), ileum AR-HR (**c**), cecum AR-HR (**d**) (n=8).
